# Applying Probability Theory for the Quality Assessment of a Wildfire Spread Prediction Framework Based on Genetic Algorithms

**DOI:** 10.1155/2013/728414

**Published:** 2013-12-25

**Authors:** Andrés Cencerrado, Ana Cortés, Tomàs Margalef

**Affiliations:** Computer Architecture and Operating Systems Department, Autonomous University of Barcelona, Bellaterra, 08193 Barcelona, Spain

## Abstract

This work presents a framework for assessing how the existing constraints at the time of attending an ongoing forest fire affect simulation results, both in terms of quality (accuracy) obtained and the time needed to make a decision. In the wildfire spread simulation and prediction area, it is essential to properly exploit the computational power offered by new computing advances. For this purpose, we rely on a two-stage prediction process to enhance the quality of *traditional* predictions, taking advantage of parallel computing. This strategy is based on an adjustment stage which is carried out by a well-known evolutionary technique: Genetic Algorithms. The core of this framework is evaluated according to the probability theory principles. Thus, a strong statistical study is presented and oriented towards the characterization of such an adjustment technique in order to help the operation managers deal with the two aspects previously mentioned: time and quality. The experimental work in this paper is based on a region in Spain which is one of the most prone to forest fires: *El Cap de Creus*.

## 1. Introduction

As stated in [1], the potential for a natural hazard to become a disaster mainly depends on a society's capacity to address the underlying risk factors, to reduce the vulnerability of a community, and to be ready to respond in case of emergency. In the last years, the scientific community has provided many advances in order to deal with the issue of the early remote sensing [2–5], which represents a great advantage. However, most of these phenomena present an additional important problem regarding the uncertainty of the variables that describe the scenario where they take place. Forest fires are a clear example of this problem, making them a very complex system to model and simulate.

Over the last decades, different physical models have been developed and implemented into forest fire spread simulators. The Rothermel model [6] can be considered the most used and representative among them. The research studies regarding fire behavior are very valuable in order to develop and optimize computational methods so as to develop simulation and prediction tools, such as FireLib [7], FireStation [8], or FARSITE [9]. Furthermore, given the complexity of these implemented models as well as their implicit computational demands, there are different works that focused on the optimization of their results either by coupling different models [10] or by exploiting the increasing computational capabilities [11, 12].

Fire spread simulators usually require input parameters that in some cases are uniform on the whole terrain and constant on time, but some others vary from one point of the terrain to another one or present a temporal evolution. Furthermore, it is very difficult to gather accurate and reliable values of certain parameters at the right places where the catastrophe is taking place, because the hazard itself often distorts the measurements. So, in many cases the unique alternative consists of working with interpolated, outdated, or even absolutely unknown values. Obviously, this fact adds the problem of dealing with high levels of uncertainty in the input parameters, which results in a lack of accuracy and quality on the provided predictions.

To overcome the input data uncertainty, a two-stage prediction methodology was developed [13]. This methodology divides the prediction process into two different stages: adjustment and prediction. During the first stage, a calibration method is applied using data about the observed fire propagation to obtain the input data set which best describes the actual evolution of the catastrophe. Afterwards, the prediction stage starts a new simulation using the values obtained in the calibration stage. Subsequently, the differences between the classical prediction schema and the two-stage prediction method are detailed.

### 1.1. Classical Prediction

**Figure 1:**
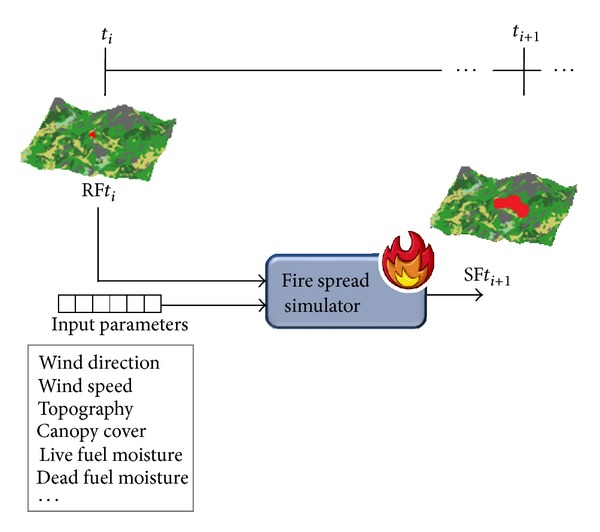
Classical prediction schema. RF and SF stand for *real fire* and *simulated fire*, respectively.

The traditional way of predicting forest fire behavior takes the initial state of the fire front as input as well as the input parameters given for that time instant. These values are entered into any existing fire simulator, which then returns the prediction for the state of the fire front at a later time instant. This is summarized in Figure 1, where *t*_*i*_ stands for a certain time instant *i*, RF*t*_*i*_ stands for the real fire spread at a certain time instant *i*, and SF*t*_*i*_ stands for the simulated spread at a certain time instant *i*.

The forecasted fire front tends to differ from the real fire line to a greater or lesser extent. As the prediction error accumulates gradually as the prediction time advances, deviations between real phenomenon behavior and forecasted fire spread become even more significant. One reason for this incidence is that the classic calculation of the simulated fire is based upon one single set of input parameters afflicted with many inadequacies. To improve parameter quality and enable real-time estimation and calibration of model input parameters in each time step during an ongoing prediction, a two-stage prediction scheme was proposed in [13].

### 1.2. Two-Stage Prediction Method

Fire spread simulators need certain input data which define the characteristics of the environment where the fire is taking place in order to evaluate its future propagation. This data usually consists of the current fire front, terrain topography, vegetation type, and meteorological data such as humidity, wind direction, and wind speed. Some of this data could be retrieved in advance and with notable accuracy, for example, the topography of the area and the predominant vegetation types.

However, there is some data that turns out to be very difficult to be reliably obtained. For instance, getting an accurate fire perimeter is very complicated because of the difficulties involved in getting real-time images. Live and dead fuel moistures are examples of data which cannot be retrieved with reliability at the moment of the emergency. Another kind of data sensitive to imprecisions is that of meteorological data, which is often distorted by the fire itself. However, this circumstance is not only related to forest fires but also happens in any system with a dynamic state evolution over time, for example, floods [14], thunderstorms [15, 16], and so forth. These uncertainties, added to the fact that these inputs are set up only at the very beginning of the simulation process, become an important drawback because, as the simulation time goes on, variables previously initialized could change dramatically, misleading simulation results. In order to overcome these restrictions, we need a system capable of properly estimating the values of the input parameters needed by the underlying simulator so that the results we obtain correspond to reality.

**Figure 2:**
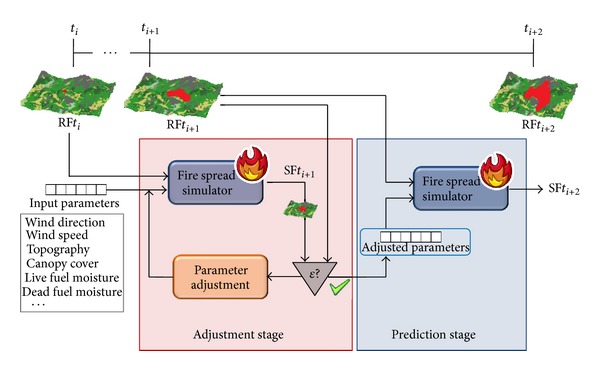
Two-stage prediction method.

Introducing a previous adjustment step, the set of input parameters is optimized before every prediction step.  Figure 2 schematizes this process. In this figure, the *ɛ* process consists of the comparison between the actual and the simulated fire spreads. As it is explained in Section 3, for this calculation we rely on the *symmetric difference* between sets of burned cells.

Thus, the proposed solution comes from reversing the problem: how to find a parameter configuration such that, given this configuration as input, the fire simulator would produce predictions that match the actual fire behavior.

Having detected the simulator input that best describes current environmental conditions, the same set of parameters could also be used to best describe the immediate future, assuming that meteorological conditions remain constant during the next prediction interval. Then, the prediction becomes the result of a series of automatically adjusted input configurations.

The process of input parameters adjustment can be seen as a global optimization process, where we search for an instance in an N-dimensional space (N equals the number of input parameters to be adjusted) which minimizes the difference between the simulated fire spread produced and the real observed spread. There is a great variety of works which rely on the so-called Global Optimization Algorithms [17] to solve different problems of this kind [18–21]. In the specific case of forest fire spread, several adjustment techniques have been tested to calibrate the input parameter set, from which stands out the use of bioinspired evolutionary computation, specifically Genetic Algorithms (GAs), which has provided outstanding results as a parallel adjustment technique within the two-stage prediction framework [22].

In order to be useful, any evolution prediction of an ongoing hazard must be delivered as fast as possible in order not to be outdated. When relying on the two-stage prediction method, accuracy also depends on the amount of computational resources we have access to performing different simulations, since the use of parallel computation favors the evolutionary methods used in the adjustment stage. As it will be studied in Section 4, the fact that the adjustment strategies for parameter optimization may be carried out in a parallel way allows us to take advantage of the simultaneous execution of different simulations.

The rest of this paper is organized as follows. In the next section, we explain the proposed methodology to assess in advance the quality of the predictions. In Section 3, the details concerning the characterization of the GAs as adjustment strategy, based on the principles of probability theory, are given. Section 4 presents a statistical study based on the area of *El Cap de Creus *(northeast of Spain) in order to validate our propose methods. Finally, the main conclusions are given in Section 5.

## 2. Methodology for Time Response and Quality Assessment for Forest Fire Spread Prediction

From the point of view of attending an ongoing emergency, it is necessary to be able to assess, in advance, the amount of computational resources needed to deal with. This is due to the existing strict deadlines for giving a response. Moreover, it is also necessary to accurately assess the quality that the prediction system will give us, since this kind of emergency may threaten urban areas and even human lives.

Based on the two-stage prediction framework, some previous studies were carried out in order to choose the most suitable adjustment strategy [23, 24]. GAs turned out to be the most appropriate technique among other Global Optimization Algorithms (such as *Tabu Search* and *Simulated Annealing*,) not only for their outstanding results but also because their nature favors parallel computing. The use of such an iterative adjustment technique implies that it leads us to the desired solution progressively, that is, the more the iterations we are able to perform, the better the solution we will be able to find. Obviously, this fact has a direct impact on the time incurred in the prediction process. So, in order to reach a good trade-off between quality and urgency, one must consider three main interrelated issues.

- The quality of the prediction is directly related to the quality of the adjustment, and the quality of the adjustment depends on how many times the adjustment technique is iterated, as well as on the number of scenarios tested per iteration.
- The amount of computing resources determines the amount of simulations that can be simultaneously executed per iteration at the adjustment stage.
- The response time in providing a prediction is a critical point and seriously limits the number of iterations that can be executed at the adjustment stage.

The solution proposed is conceived to fulfill the necessity of deploying a way to set up in advance:

- the prediction scheme settings, in particular the adjustment policy's specific parameters, for a required quality of the prediction; this is especially relevant when the ongoing fire may threaten urban areas and even human lives;
- the computational resources needed to deliver a required quality of the prediction, given certain time constraints.

In order to properly tackle this objective, the details of the proposed methodology for the characterization of the two-stage prediction method are given in the subsequent subsection.

### 2.1. Two-Stage Prediction Method Characterization

Based on the assumptions and requirements detailed above, we have designed a methodology to characterize the two-stage prediction process. For this methodology to be as flexible as possible (simulator-independent), we have to analyze the behavior of the aforementioned processes in terms of the main variables that we must deal with: the time spent and the quality of the results.  Table 1 summarizes the dependencies for each case. As one can see, there is a series of dependencies from the prediction quality to the simulation time.

As regards the time needed for the final prediction process, it consists of a single simulation of the winning input setting at the adjustment stage, and the quality of this simulation is directly correlated to the quality obtained at the end of the adjustment process [25].

Since the developed adjustment methods are all iterative, the quality obtained at the end of the adjustment stage presents a single dependence: the time available to perform it. Regarding the necessary time, this process presents a couple of dependencies; obviously, the time incurred in each simulation will determine the overall adjustment time, but it also depends on the specific configuration of the adjustment method itself. Since the adjustment strategies may be run in a parallel way, in many cases this configuration can take greater advantage of the available resources. Thus, the time incurred in the adjustment process will also depend on the available computational resources.

Finally, each simulation, in terms of time needed, is dependent on the input parameters that describe the scenario being simulated and the underlying computational resources where it is executed. The quality of each simulation will depend on the input parameters, since it is determined by the similarity between the simulation run using those input parameters and the actual evolution of the fire. Therefore, the characterization must be done from the simulation process to the prediction process, so that by means of characterizing the time incurred in the simulation process in terms of its inputs and the computational resources, we can reach the final prediction assessment.

To accomplish this goal, it is necessary to characterize the adjustment strategy in such a way that it is possible to determine beforehand the number of iterations and the number of scenarios per iteration that should be executed to ensure a certain prediction quality. Since each scenario implies one execution of the simulation kernel, it is also necessary to characterize this simulation kernel to estimate the time required to run each simulation. For this reason, we rely on a methodology for classifying the scenarios according to how long their simulation will take before their execution, based on the use of Decision Trees [26] as classification strategy. This methodology is widely discussed in previous works, such as [27, 28], and allows us to carry out a premature detection of lengthy simulations so that we can avoid their inclusion in the adjustment process. As it will be seen in Section 4, this represents a very important advantage for the success of our proposed methods.

Having characterized the adjustment technique and the simulation time, then it is possible to determine the necessary computing resources to execute a certain number of iterations with a certain number of simulations per iteration. Thus, our methodology allows us to determine the required computing resources to reach a certain prediction quality in a given time.

## 3. Genetic Algorithm Characterization

By their own nature, GAs constitute a technique that works in an iterative way; that is, the quality of its results directly depends on the times it is iterated as well as its specific configuration settings.

It starts with an initial population of individuals which will be evolved over several iterations in order to guide them to better search space areas. The individuals used in the case of forest fire spread prediction are defined as a sequence of different genes, namely, wind speed and wind direction, moisture content of the live fuel, moisture content of the dead fuel (at three different times), and type of vegetation, out of the 13 standard Northern Forest Fire Laboratory fuel models [29]. Topographic data is constant, so it is not considered in the evolutionary process.

Operators such as *elitism*,* selection*, *crossover*, and *mutation* are applied to every population to obtain a new one superior to the previous one. Elitism consists of keeping the best individuals from one generation to the next one. By this way, it is guaranteed that the solution coming from a certain generation is, at least, as good as the one obtained from the previous generation. Selection operation selects good quality parents to create children that will inherit their parents' good characteristics (by crossover operation). In order to guarantee natural diversity of individual characteristics, mutation can occur for each child characteristic (under a very slight probability).

Each individual from a population is ranked according to a predefined fitness function. Since simulated fires can be represented as a grid of cells map, indicating which cells were burned as a consequence of the simulated fire, in our case the quality of a specific individual is determined by means of the fitness function expressed by (1). This equation calculates the differences in the number of cells burned, both missing or in excess, between the simulated and the real fire. Formally, this formula corresponds to the *symmetric difference* between the actual spread and the simulated spread, divided by the actual spread, so as to express a proportion. *UnionCells* is the union of the number of cells burned in the real fire and the cells burned in the simulation, *IntersecCells* is the intersection between the number of cells burned in the real fire and in the simulation, *RealCells* are the cells burned in the real fire, and *InitCells* are the cells burned at the starting time:

(1) Error =(UnionCells−InitCells)−(IntersecCells−InitCells)RealCells−InitCells.

The iterative nature of GAs leads to a near optimal solution in the adjustment stage after a certain number of iterations. For this reason, it is mandatory to analyze the GA convergence for the particular case of forest fire spread prediction, as well as to be able to extract a general characterization of its behavior.

The characterization of GAs as an adjustment technique for the two-stage prediction method must allow us to properly estimate the adjustment quality we may obtain, given certain restrictions, both in terms of timing and resource availability when the adjustment stage is done. Adjustment quality stands for the difference between the simulation result once the adjustment process is completely carried out and the real state of the hazard.

In general terms, this issue is addressed by means of performing a statistical analysis to determine, for each adjustment strategy, the features that affect the quality of the results. Then, a study of the particular impact of these factors on the convergence of each method is done, to infer criteria in order to estimate the achievable quality of results under certain restrictions.

Parameters, such as number of generations, individuals per population, elitism factor, and mutation probability, affect the quality of the winner individual, that is, the final solution we will deliver at the end of the adjustment process.

### 3.1. GA Characterization Methodology

Thus, our proposed methodology for the GA characterization can be summarized in the following steps.

1. GA key settings identification: it is necessary to assess and determine the settings that could affect the quality of the results when using GA as the adjustment technique. So far, the analyzed factors in this work are the size of the populations, the number of generations, and the mutation probability.
2. Study of the impact on convergence: depending on the specific case (mainly, the topographic area), the influence of certain GA settings upon the quality of the results may be decreased in favor of others and vice versa. Therefore, it is necessary to study the particular impact of these factors on the convergence for each case.
3. Statistical analysis: this analysis consists of identifying which probability distribution best fits the results obtained, in terms of the quality of the adjustment obtained. Then, by means of the corresponding probability density function, we are able to establish a guarantee degree in our estimations, as well as determine which configuration of the GA is most suitable.

These steps are made *offline*, which means that they are all already carried out at the moment of the fire occurrence. It is also worth mentioning that the results and applicability of this methodology are independent of the features of the computational platforms being used.

Having completed these three processes, we are ready to infer criteria to determine the achievable adjustment quality under certain restrictions and are therefore able to advise on the best specific settings for the GA.

## 4. Experimental Study: GA Characterization Based on *Cap de Creus* Landscape

**Figure 3:**
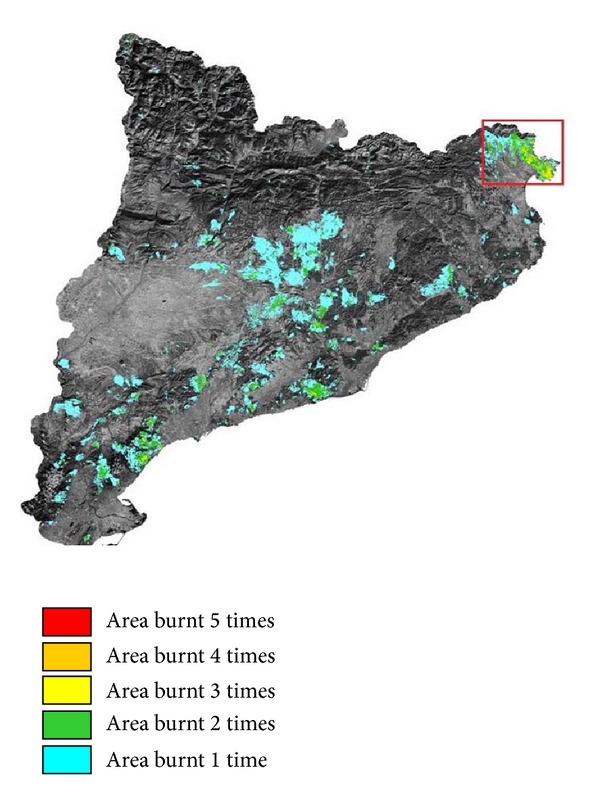
Fire occurrences in Catalonia in the period 1975–2010 (fire sizes bigger than or equal to 30 ha).

**Figure 4:**
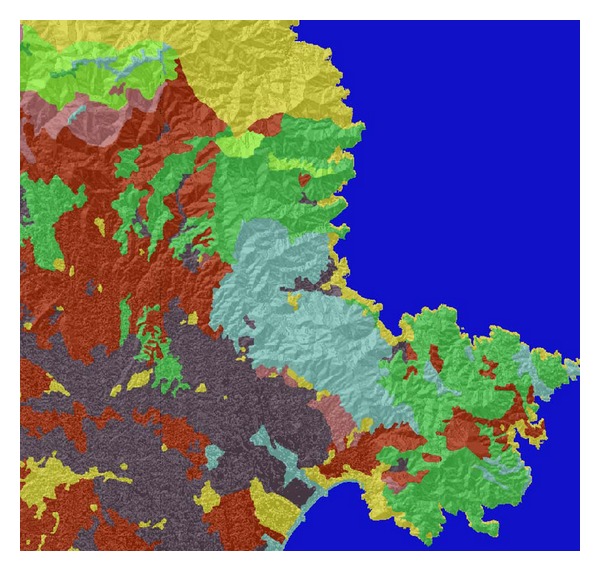
*Cap de Creus*, Catalonia (image from FARSITE simulator). Different colors indicate different types of vegetation.

The experimental study presented in this work is based on one of the most problematic areas in Catalonia (northeast of Spain).  Figure 3 shows the occurrences of fires bigger than 30 hectares in the period 1975–2010 [2, 30]. The most recurrent area corresponds to the northeast cape (*El Cap de Creus*). This area is zoomed in in Figure 4 and has an approximate real extension of 900 square kilometers.

Subsequently, we test our methodology using this landscape. The fire spread simulator used was FARSITE [9].

### 4.1. GA Statistical Study

The analysis of the convergence and the statistical study were performed in the following terms:

- populations composed of 25 and 100 individuals;
- populations evolved over 10 generations;
- mutation probability: 10%;
- crossover probability: 70%;
- initial fire consisting of a single initial ignition point;
- adjustment time interval: 0 h–6 h (from the ignition to 6 hours after).

Using these configurations for the GA, we carried out the evolution process for 100 different populations.

From the obtained results, a statistical study carrying out the Kolmogorov-Smirnov, Anderson-Darling, and Chi-squared tests allowed us to determine that the probability distribution which best fits the obtained data, regarding the obtained quality of adjustment, is the *Lognormal* distribution, which is a continuous probability distribution of a random variable, whose logarithm is normally distributed. Its probability density function (PDF) is as follows:

(2) $$\begin{array}{c} {\text{pdf}\left(x;\mu ,\sigma \right)=\frac{1}{x\sigma \sqrt{2\pi }}e}^{-{\left(\ln x-\mu \right)}^{2}/2\sigma^{2}}\;\;\;x>0. \end{array}$$

In this equation, *x* is the random variable (which corresponds to the error obtained at the end of the adjustment process), *μ* is the mean, and *σ* is the standard deviation of the variables natural logarithm (by definition, the variables logarithm is normally distributed).

**Figure 5:**
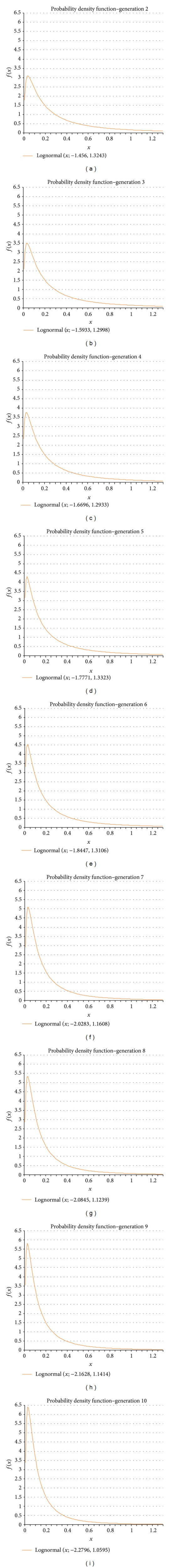
Probability density functions for the obtained errors at each generation of the evolution process.

Although the probability distribution of the data is the same throughout the whole evolution process, these factors vary depending on the iteration of the GA we are evaluating. So, Figure 5 depicts the different PDFs for each generation, for the particular case of 25-individual populations.

By means of these PDFs, we can guarantee the maximum adjustment error we will obtain, given a certain configuration of the GA, with different degrees of certainty. In addition, since the number of generations has a direct impact on both the available resources and the time needed to perform the adjustment process, it is worth highlighting that we are able to give this guarantee taking into account the number of generations we are able to execute.

For instance, Tables 2 and 3 show the different maximum adjustment errors (considering the adjustment time interval [0 hours–6 hours]) for which we have different *guarantee degrees* in the cases of 25 and 100 individuals per population, respectively. Here, *guarantee degree* stands for the probability of obtaining an adjustment error lesser than or equal to the specified value, on the basis of the above presented PDF (2).

**Figure 6:**
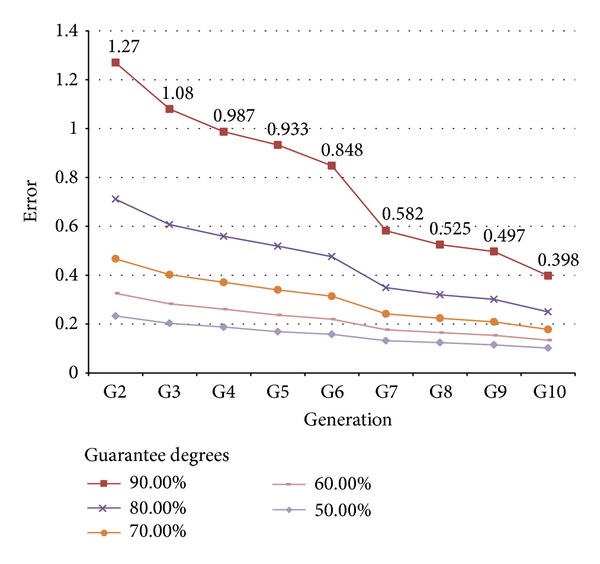
Achievable error with different degrees of guarantee. Populations composed of 25 individuals.

**Figure 7:**
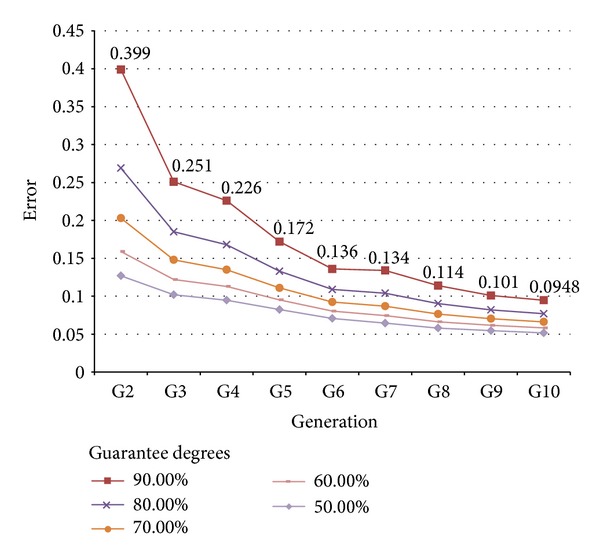
Achievable error with different degrees of guarantee. Populations composed of 100 individuals.

Figures 6 and 7 also depict this information, from a guarantee degree of 90% down to 70%. As can be easily understood, the lesser the error requested is, the lesser the degree of guarantee, for the same number of iterations of the GA.

Considering a real situation, where the quality of the prediction is a parameter fixed by the decision control centre in charge of making the appropriate decisions, this information turns out to be very important, since we are able to give a certain guarantee of quality in the final prediction, taking into account how many evolution steps (i.e., how many generations) we can perform. Moreover, it is also possible to fix the quality of the adjustment and then determine the guarantee degree of reaching such an error in a given number of iterations.

### 4.2. Real Emergency Recreation

In order to prove the correctness of the above presented characterizations, we consider a hypothetical situation based on the *Cap de Creus* landscape, where we have to meet the following restrictions regarding both the quality of the adjustment and the time available to perform it.

- Response time: one hour for the adjustment process.
- Quality of adjustment: a maximum error of approximately 0.5 is required.

As stated in Section 2.1, we rely on a Decision Tree-based method which allows us to rapidly assess the simulation time a certain configuration of the scenario will produce, without the necessity of executing it [27, 28]. Therefore, by means of the premature detection and discard of lengthy simulations, we are able to limit the elapsed time in the adjustment stage.

For the particular case of this work, we applied this method to allow the inclusion of individuals whose simulations do not exceed 750 seconds (this detection process presented a hit ratio of 96.4% using a test set of 1000 individuals randomly generated). By this way, assuming that we have enough computational resources so as to execute 25 simulations in parallel (25 computing cores), we are able to evolve a population composed of 25 individuals over 5 generations in 3750 seconds (1 hour, 2 minutes, and 30 seconds), which would be appropriate.

By analyzing the data in Table 2, we assume that we could bring an error approximately equal to 0.5 with an 80% degree of guarantee at the end of the fifth generation (the error indicated is 0.519, row “80%”, column “G5”).

So, we carried out an experiment consisting of the evolution of 30 populations composed of 25 individuals. At the fifth generation, we should obtain an error equal to or lesser than 0.519 in approximately 80% of cases.

Table 4 shows the results obtained from these evolutions. These evolution processes were extended up to 10 generations for further analysis purposes. As can be seen, only 3 populations (p1, p19, and p22) out of 30 exceeded an error of 0.5 at the end of the fifth generation, which goes beyond the assumed 80% degree of guarantee. Moreover, by limiting the time involved in the adjustment process to 750 seconds, the response time restriction was also met.

Indeed, we can observe that in this experimental set only populations p19 and p22, in some generations, exceeded the errors corresponding to 90% degree of guarantee (see Table 2), which turns out to be an absolutely satisfactory result.

## 5. Conclusions

The novelty of our approach is the design of a methodology for the early assessment of both the time needed to perform a prediction and its quality within the proposed two-stage prediction framework, focusing on the specific case of forest fire spread prediction.

In the field of fire spread simulation and prediction, dealing with high degrees of uncertainty on the input parameters is common, which may lead to important losses in the quality of the predictions. The two-stage prediction scheme was introduced to relieve the problem of such input parameter uncertainty. This prediction framework highly improves the quality of the predictions but, due to its complexity, it could be very hard to know (or even to estimate) how much time is necessary in such processes, as well as the ideal amount and type of computational resources to be used.

Given the high level of uncertainty and complexity in the underlying scenario that describes the initial conditions of the catastrophe, it is necessary to rely on the so-called Global Optimization Algorithms to improve the quality of the predictions. The performance of these kinds of algorithms, however, may present high variability due to their specific initial settings. This variability may have impact on the quality of the results as well as the time needed to carry out the optimization process. For this reason, we present our methodology to characterize a well-known Artificial Intelligence technique, Genetic Algorithms, which has proven to be a powerful technique for performing the adjustment process in our two-stage prediction method. Thus, we relied on the probability theory area to carry out a statistical study based on a huge set of fire spread simulations using the *Cap de Creus* landscape as a real case study. Then, we have identified the probability distribution which corresponds to the results obtained, so that we can rely on its probability density function in order to establish certain degrees of guarantee in accuracy estimations.

This methodology allows us to keep to the occasional existing limitations as regards the time available to provide a prediction and the amount of computing resources we can access. This represents a great advantage for the forest fire operation management teams.

The experimental study presented highlights the suitability of the proposed methods, which can be easily extrapolated in order to be applied in management actions related to other kinds of natural hazards.

## Figures and Tables

**Table 1:** Dependencies between each factor belonging to the two-stage prediction framework.

Simulation		Adjustment		Final prediction	
Time	Quality	Time	Quality	Time	Quality
Input settings	Input settings	Simulation time	Adjustment time	Simulation time	Adjustment quality and real-time eventualities
Computational resources		Computational resources			
		Configuration of the adjustment method			

**Table 2:** Achievable error with different degrees of guarantee (populations composed of 25 individuals).

Guarantee degree	G2	G3	G4	G5	G6	G7	G8	G9	G10
95%	2.06	1.72	1.58	1.51	1.36	0.888	0.79	0.752	0.585
90%	1.27	1.08	0.987	0.933	0.848	0.582	0.525	0.497	0.398
85%	0.92	0.782	0.719	0.673	0.615	0.438	0.399	0.357	0.307
80%	0.711	0.607	0.559	0.519	0.476	0.349	0.32	0.301	0.25
75%	0.57	0.488	0.45	0.415	0.383	0.288	0.265	0.248	0.209
70%	0.467	0.402	0.371	0.34	0.314	0.242	0.224	0.209	0.178
65%	0.388	0.335	0.31	0.283	0.262	0.206	0.192	0.179	0.154
60%	0.326	0.283	0.261	0.237	0.22	0.177	0.165	0.154	0.134
55%	0.275	0.239	0.222	0.2	0.186	0.152	0.143	0.133	0.117
50%	0.233	0.203	0.188	0.169	0.158	0.132	0.124	0.115	0.102

**Table 3:** Achievable error with different degrees of guarantee (populations composed of 100 individuals).

Guarantee degree	G2	G3	G4	G5	G6	G7	G8	G9	G10
95%	0.552	0.325	0.289	0.211	0.164	0.164	0.138	0.121	0.113
90%	0.399	0.251	0.226	0.172	0.136	0.134	0.114	0.101	0.095
85%	0.32	0.212	0.191	0.149	0.12	0.116	0.1	0.09	0.085
80%	0.269	0.185	0.168	0.133	0.109	0.104	0.09	0.082	0.077
75%	0.232	0.164	0.15	0.121	0.099	0.095	0.083	0.076	0.071
70%	0.203	0.148	0.135	0.111	0.092	0.087	0.077	0.07	0.066
65%	0.179	0.134	0.123	0.103	0.086	0.08	0.071	0.066	0.062
60%	0.159	0.122	0.113	0.095	0.08	0.074	0.067	0.062	0.058
55%	0.142	0.112	0.103	0.089	0.075	0.069	0.062	0.058	0.055
50%	0.127	0.102	0.095	0.082	0.071	0.064	0.058	0.055	0.052

**Table 4:** Errors obtained from the evolution of 30 populations composed of 25 individuals in *Cap de Creus* landscape.

Population	G2	G3	G4	G5	G6	G7	G8	G9	G10
p0	0.447	0.414	0.265	0.265	0.124	0.124	0.08	0.08	0.072
p1	0.673	0.673	0.673	0.614	0.164	0.164	0.164	0.164	0.164
p2	0.251	0.251	0.251	0.18	0.025	0.025	0.025	0.025	0.025
p3	0.445	0.067	0.067	0.067	0.067	0.067	0.067	0.067	0.067
p4	0.235	0.125	0.099	0.035	0.035	0.035	0.019	0.019	0.005
p5	0.601	0.455	0.45	0.427	0.139	0.139	0.139	0.139	0.139
p6	0.417	0.417	0.417	0.246	0.246	0.219	0.12	0.12	0.105
p7	0.518	0.055	0.055	0.055	0.055	0.038	0.038	0.033	0.018
p8	0.716	0.411	0.122	0.061	0.044	0.044	0.044	0.044	0.044
p9	0.567	0.552	0.494	0.491	0.49	0.49	0.488	0.488	0.488
p10	0.742	0.694	0.59	0.353	0.351	0.351	0.351	0.351	0.351
p11	0.453	0.147	0.147	0.125	0.125	0.095	0.095	0.058	0.054
p12	0.31	0.31	0.31	0.31	0.241	0.234	0.097	0.096	0.096
p13	0.565	0.174	0.174	0.122	0.122	0.122	0.115	0.115	0.111
p14	0.341	0.339	0.223	0.223	0.217	0.217	0.217	0.117	0.117
p15	0.388	0.257	0.117	0.061	0.061	0.061	0.061	0.038	0.038
p16	0.75	0.414	0.272	0.272	0.248	0.248	0.245	0.179	0.038
p17	0.603	0.218	0.218	0.218	0.212	0.169	0.169	0.15	0.134
p18	0.507	0.48	0.342	0.224	0.22	0.197	0.197	0.197	0.034
p19	1.221	1.201	1.201	0.995	0.995	0.995	0.728	0.624	0.444
p20	0.448	0.448	0.448	0.23	0.23	0.17	0.17	0.17	0.092
p21	0.758	0.758	0.407	0.407	0.407	0.126	0.126	0.126	0.087
p22	1.01	1.01	1.01	1.01	0.876	0.612	0.612	0.312	0.259
p23	0.108	0.108	0.108	0.108	0.108	0.105	0.105	0.071	0.071
p24	0.214	0.111	0.111	0.111	0.104	0.104	0.104	0.086	0.086
p25	0.303	0.175	0.172	0.172	0.109	0.109	0.109	0.091	0.091
p26	0.637	0.549	0.444	0.042	0.039	0.037	0.037	0.037	0.032
p27	0.338	0.338	0.099	0.099	0.099	0.099	0.099	0.092	0.092
p28	0.75	0.51	0.155	0.155	0.066	0.066	0.066	0.066	0.016
p29	0.57	0.461	0.425	0.193	0.193	0.096	0.096	0.096	0.096
